# Personal reflections on the neuropathology of kuru

**DOI:** 10.1098/rstb.2008.4014

**Published:** 2008-11-27

**Authors:** Byron A. Kakulas

**Affiliations:** Australian Neuromuscular Research Institute, University of Western AustraliaCrawley, WA 6009, Australia

My involvement with kuru began towards the end of 1963 while I was a Fellow at the Massachusetts General Hospital (MGH). During the autumn of that year I was contacted by Dr Michael Alpers, who was on his way to Boston from the National Institutes of Health (NIH) in Bethesda, Maryland with a carload of New Guinean artefacts to be delivered to the Peabody Museum in Salem, Massachusetts on behalf of Dr Carleton Gajdusek. We had been students together at Adelaide University and St Mark's College in the 1950s and our friendship was thus rekindled.

Sometime after this I was approached by representatives of NIH to investigate the Parkinsonism–dementia (PD) and amyotrophic lateral sclerosis (ALS) neurological complex prevalent on Guam, where it was the main cause of death among the Chamorro people. At that time, the cause of this devastating neurological complex was subject to a great deal of conjecture. These discussions led to visits to Bethesda, where I met Carleton and Joe Gibbs. This was at the exciting time when the first chimpanzees developed symptoms of kuru. Lifetime friendships were thus established with Carleton and Joe and with Jake Brody, as well as with Dr Asao Hirano in New York, who was the first to describe the neuropathology of the Guam disorders.

As I was committed to the University of Western Australia, being on leave from an academic post in the medical school and feeling that I had a mission to set up neuropathology in my home country, I was not able to pursue the very attractive Guam opportunity and returned to Perth in 1965.

However, earlier in that year, while deeply involved in a very busy schedule at Harvard Medical School working with my mentor in muscle diseases Dr Raymond D. Adams, Chief of Neurology at MGH, I was approached by an enthusiastic French-Canadian neurologist André-Roche Lecours. He told me about two kuru brains that had been cut in serial section at the Warren Museum of Harvard Medical School under the watchful eye of the distinguished developmental neuroscientist Dr Paul Yakovlev. Roche had concluded that I had the expertise to study and report on the two brains in association with him.

At this time I had a large number of research projects going as well as being Chief Resident in Neuropathology under Dr Pierson Richardson at MGH, so I had no time at all for such painstaking work. Nevertheless, Roche prevailed and we both gave up our Sunday mornings for several weeks to examine the 5000 slides of each of the two kuru brains. Our wives were not too pleased.

This investigation resulted in what may be considered the definitive report of the neuropathology of kuru (Kakulas *et al.* 1967). In this work we were able to show that the severity of the lesions was greatest in the limbic lobes of the brain and in the vermis of the cerebellum, thus demonstrating a very strong clinical correlation between the lesions and the symptoms. Vermal atrophy underlay the cerebellar ataxia and the dementia was due to the hippocampal and related limbic zones being most affected.

Later, in Australia, I had the privilege of further collaboration with Michael and Carleton, having been sent a large number of New and Old World primate brains to work up neuropathologically. The purpose of these studies was to establish incubation periods and species susceptibility for both kuru and Creutzfeldt–Jakob disease (CJD). By this time, Colin Masters had returned to Perth as a National Health and Medical Research Council (NH&MRC) Fellow and he joined the studies. These investigations resulted in the unexpected finding that typical kuru and CJD changes were well established in these brains before symptoms appeared (Masters *et al.* 1976) thus demonstrating a high degree of ‘cerebral reserve’, which neuropathologists had theoretically suggested existed.

As a matter of interest I was also asked to report on a large number of Guamanian ALS–PD cases by the NIH authorities. I was joined by Prof. Henry Urich in these studies and later by Dr Dan Perl. Not only did these investigations establish a large overlap in the distribution of lesions between the three entities but also consolidated the toxic cycad hypothesis as being the cause of the neurological degeneration underlying the Guam conditions. Although the affected Chamorro patients clinically manifested mainly either Parkinsonism–dementia on the one hand or ALS on the other, the lesions were found to be much more widespread in most patients and involved the spinal cord, midbrain and cerebral cortex in each, but to a varying degree, thus demonstrating again a preclinical ‘incubation’ period and a threshold effect prior to symptoms and signs occurring.

Incidentally, I should mention that all this work took place in parallel with my main interest in muscle disease and spinal injuries. My gratitude is expressed to the organizers for the opportunity to participate in the End of Kuru meeting.
